# Supplementary material for: Determinants of burnout and other aspects of psychological well-being in healthcare workers during the Covid-19 pandemic: A multinational cross-sectional study
Source: PLoS One. 2021 Apr 16;16(4):e0238666. doi: 10.1371/journal.pone.0238666 (PMC8051812; doi:10.1371/journal.pone.0238666)
Supplement: S1 File — A: English COVID-19 Questionnaire. B: Polish COVID-19 Questionnaire. C: SAQ Scoring. (DOCX) [file pone.0238666.s001.docx]

**APPENDIX A. ENGLISH COVID-19 QUESTIONNAIRE**

**About You**

| 1.1 | Age |
| --- | --- |
| 1.2 | Gender |
| 1.3 | Ethnicity - British census category (Optional) |
| 1.4 | Role |
| 1.5 | Where are you based? |
| 1.6 | Specialty or area that you were working in prior to the COVID pandemic? |
| 1.7 | Have you been redeployed to a different area, hospital, or specialty? |
| 1.8 | If so, to which specialty or area have you been redeployed? |
| 1.9 | If redeployed: how would you describe the training you received in preparation for redeployment? |
| 1.10 | If redeployed: how would you describe the support you received during redeployment? |
| 1.11 | How many days did you spend at work in the last 7 days? |
| 1.12 | What was the average duration of your shift? |
| 1.13 | Approximately how many cases of suspected or confirmed COVID-19 are in your hospital or place of work? |
| 1.14 | Have you treated a COVID-19 positive patient in the past week? |
| 1.15 | In the past week, what is your own COVID-19 status? |
| 1.16 | I work in a |
| 1.17 | (Optional) Current Hospital/Trust |

**Safety Attitudes Questionnaire**

| 2.1 | Nurse input is well received in this clinical area. |
| --- | --- |
| 2.2 | In this clinical area, it is difficult to speak up if I perceive a problem with patient care. |
| 2.3 | Disagreements in this clinical area are resolved appropriately (i.e., not who is right, but what is best for the patient). |
| 2.4 | I have the support I need from other personnel to care for patients. |
| 2.5 | It is easy for personnel here to ask questions when there is something that they do not understand. |
| 2.6 | The physicians and nurses here work together as a well-coordinated team. |
| 2.7 | I would feel safe being treated here as a patient. |
| 2.8 | Medical errors are handled appropriately in this clinical area. |
| 2.9 | I know the proper channels to direct questions regarding patient safety in this clinical area. |
| 2.10 | I receive appropriate feedback about my performance. |
| 2.11 | In this clinical area, it is difficult to discuss errors. |
| 2.12 | I am encouraged by my colleagues to report any patient safety concerns I may have. |
| 2.13 | The culture in this clinical area makes it easy to learn from the errors of others. |
| 2.14 | My suggestions about safety would be acted upon if I expressed them to management. |
| 2.15 | I like my job. |
| 2.16 | Working here is like being part of a large family. |
| 2.17 | This is a good place to work. |
| 2.18 | I am proud to work in this clinical area. |
| 2.19 | Morale in this clinical area is high. |
| 2.20 | When my workload becomes excessive, my performance is impaired. |
| 2.21 | I am less effective at work when fatigued. |
| 2.22 | I am more likely to make errors in tense or hostile situations. |
| 2.23 | Fatigue impairs my performance during emergency situations (e.g. emergency resuscitation, seizure). |
| 2.24 | Management supports my daily efforts. |
| 2.25 | Management doesn’t knowingly compromise patient safety. |
| 2.26 | Management is doing a good job. |
| 2.27 | Problem personnel are dealt with constructively by our management. |
| 2.28 | I get adequate, timely info about events that might affect my work, from management. |
| 2.29 | The levels of staffing in this clinical area are sufficient to handle the number of patients. |
| 2.30 | This hospital does a good job of training new personnel. |
| 2.31 | All the necessary information for diagnostic and therapeutic decisions is routinely available to me. |
| 2.32 | Trainees in my discipline are adequately supervised. |
| 2.33 | I experience good collaboration with nurses in this clinical area. |
| 2.34 | I experience good collaboration with staff physicians in this clinical area. |
| 2.35 | I experience good collaboration with pharmacists in this clinical area. |
| 2.36 | Communication breakdowns that lead to delays in delivery of care are common. |

**Oldenburg Burnout Inventory**

| 3.1 | I always find new and interesting aspects of my work. |
| --- | --- |
| 3.2 | There are days when I feel tired before I arrive at work. |
| 3.3 | It happens more and more often that I talk about my work in a negative way. |
| 3.4 | After work, I tend to need more time than in the past in order to relax and feel better |
| 3.5 | I can tolerate the pressure of my work very well. |
| 3.6 | Lately, I tend to think less at work and do my job almost mechanically. |
| 3.7 | I find my work to be a positive challenge. |
| 3.8 | During my work, I often feel emotionally drained. |
| 3.9 | Over time, one can become disconnected from this type of work. |
| 3.10 | After working, I have enough energy for my leisure activities. |
| 3.11 | Sometimes I feel sickened by my work tasks. |
| 3.12 | After my work, I usually feel worn out and weary. |
| 3.13 | This is the only type of work that I can imagine myself doing. |
| 3.14 | Usually, I can manage the amount of my work well. |
| 3.15 | I feel more and more engaged in my work. |
| 3.16 | When I work, I usually feel energized. |

**Hospital anxiety and depression scale**

| 4.1 | I feel tense or 'wound up' |
| --- | --- |
| 4.2 | I still enjoy the things I used to enjoy |
| 4.3 | I get a sort of frightened feeling as if something awful is about to happen |
| 4.4 | I can laugh and see the funny side of things |
| 4.5 | Worrying thoughts go through my mind |
| 4.6 | I feel cheerful |
| 4.7 | I can sit at ease and feel relaxed |
| 4.8 | I feel as if I am slowed down |
| 4.9 | I get a sort of frightened feeling like 'butterflies' in the stomach |
| 4.10 | I have lost interest in my appearance |
| 4.11 | I feel restless as I have to be on the move |
| 4.12 | I look forward with enjoyment to things |
| 4.13 | I get sudden feelings of panic |
| 4.14 | I can enjoy a good book or radio or TV program |

**Any other comments**

| 5.1 | Is there anything else you would like to add or comment on, especially in relation to redeployment, wellbeing, or safety during Covid-19? |
| --- | --- |

**APPENDIX B. POLISH QUESTIONNAIRE**

**Podstawowe informacje (About You)**

| 1.1 | Wiek |
| --- | --- |
| 1.2 | Płeć |
| 1.3 | Etniczność (opcjonalne) |
| 1.4 | Rola |
| 1.5 | W którym województwie pracujesz? |
| 1.6 | W jakim zespole/na jakim oddziale pracowałeś/aś przed epidemią COVID-19? |
| 1.7 | Czy zostałeś/aś przeniesiony/a do innego zespołu lub oddziału? |
| 1.8 | Jeśli tak, to jakiego zespołu lub oddziału zostałeś/aś przeniosiony/a? |
| 1.9 | Jeżeli zostałeś/aś przeniesiony/a: jak oceniasz szkolenie przygotowujące do nowej roli? |
| 1.10 | Jeżeli zostałeś/aś przeniesiony/a: jak oceniasz wsparcie otrzymane w nowej roli? |
| 1.11 | Ile dni spędziłeś/aś w pracy w ciągu ostatnich 7 dni? |
| 1.12 | Ile godzin trwa średnio teraz Twoja zmiana? |
| 1.13 | Mniej więcej ile osób z podejrzeniem bądź potwierdzonym COVID-19 znajduje się w Twoim szpitalu/klinice/przychodni? |
| 1.14 | Czy w ciągu ostatniego tygodnia udzielałeś/aś leczenia pacjentowi z potwierdzonym COVID-19? |
| 1.15 | W ciągu ostatniego tygodnia, jaki był Twój stan dot. COVID-19? |
| 1.16 | Pracuję w: |
| 1.17 | (Opcjonalne) W jakim szpitalu pracujesz? |

**Jak bezpieczne jest Twoje miejsce pracy? (SAQ)**

| 2.1 | Wkład pielęgniarek jest dobrze przyjmowany na moim oddziale. |
| --- | --- |
| 2.2 | W moim miejscu pracy trudno jest mi „mówić głośno” o napotkanych przeze mnie problemach w opiece nad pacjentem. |
| 2.3 | Na moim oddziale kwestie sporne są rozstrzygane we właściwy sposób, tzn. nie ważne „kto” reprezentuje dany pogląd, tylko czy jest on dobry dla pacjenta. |
| 2.4 | Mam wsparcie, którego potrzebuję od innych pracowników w celu opieki nad pacjentami. |
| 2.5 | Personel może łatwo zadawać pytania, gdy czegoś nie rozumie. |
| 2.6 | W moim oddziale lekarze i pielęgniarki współpracują jak zgrany zespół. |
| 2.7 | Czułbym/abym się bezpiecznie przechodząc tutaj leczenie jako pacjent. |
| 2.8 | Na moim oddziale radzimy sobie z błędami medycznymi we właściwy sposób. |
| 2.9 | Wiem w jaki sposób i do kogo, kierować pytania dotyczące bezpieczeństwa pacjenta w moim miejscu pracy. |
| 2.10 | Otrzymuję konstruktywną informację zwrotną odnośnie wykonywanej przez ze mnie pracy. |
| 2.11 | W moim miejscu pracy trudno jest dyskutować na temat popełnianych błędów. |
| 2.12 | Jestem zachęcany/a przez moich współpracowników do zgłaszania jakichkolwiek obaw dotyczących bezpieczeństwa pacjenta. |
| 2.13 | Kultura panująca w moim oddziale sprawia, że łatwo jest uczyć się na błędach innych członków zespołu. |
| 2.14 | Moje sugestie dotyczące bezpieczeństwa pacjenta byłyby pozytywnie rozpatrzone, gdybym przedstawił/a je osobom zarządzającym. |
| 2.15 | Lubię swoją pracę. |
| 2.16 | Praca tutaj to jak bycie częścią dużej rodziny. |
| 2.17 | To dobre miejsce do pracy. |
| 2.18 | Odczuwam dumę mogąc pracować na tym oddziale. |
| 2.19 | Morale są wysokie na tym oddziale. |
| 2.20 | Przy nadmiernym nakładzie pracy moja wydajność spada. |
| 2.21 | Jestem mniej skuteczny/a w pracy, kiedy odczuwam zmęczenie. |
| 2.22 | Prawdopodobieństwo, że popełnię jakiś błąd wzrasta w napiętych lub wrogich sytuacjach. |
| 2.23 | Zmęczenie pogarsza moją skuteczność podczas nagłych wydarzeń (np. resuscytacja, napady padaczki). |
| 2.24 | Zarząd placówki wspiera moje codzienne działania. |
| 2.25 | Zarząd placówki nie narusza świadomie bezpieczeństwa pacjentów. |
| 2.26 | Zarząd wykonuje swoją pracę właściwie. |
| 2.27 | Zarząd placówki potrafi konstruktywnie poradzić sobie z problematycznymi pracownikami. |
| 2.28 | Otrzymuję na czas adekwatne informacje od zarządu o wydarzeniach, które mogą wpłynąć na moją pracą. |
| 2.29 | Liczba zatrudnionego personelu w moim oddziale/klinice jest adekwatna do liczby pacjentów. |
| 2.30 | Moja placówka dobrze radzi sobie ze szkoleniem nowego personelu. |
| 2.31 | Wszystkie potrzebne informacje dotyczące decyzji diagnostycznych i terapeutycznych są dla mnie zwykle dostępne. |
| 2.32 | Stażyści w mojej dziedzinie są odpowiednio nadzorowani. |
| 2.33 | Jestem usatysfakcjonowany/a z jakości współpracy z pielęgniarkami z mojego oddziału. |
| 2.34 | Jestem usatysfakcjonowany/a z jakości współpracy z lekarzami z mojego oddziału. |
| 2.35 | Dobrze współpracuje mi się z farmaceutami w moim obszarze klinicznym. |
| 2.36 | Często zdarza się, że błędy w komunikacji prowadzą do opóźnienia świadczeń zdrowotnych. |

**Wypalenie zawodowe (OLBI)**

| 3.1 | Zawsze odnajduję nowe i interesujące aspekty mojej pracy. |
| --- | --- |
| 3.2 | Są dni, kiedy już przed pracą czuję się zmęczony/a. |
| 3.3 | Coraz częściej zdarza się, że mówię o mojej pracy w negatywny sposób. |
| 3.4 | Po pracy zwykle potrzebuję więcej czasu niż kiedyś, aby się zrelaksować i wrócić do formy. |
| 3.5 | Bardzo dobrze znoszę presję związaną z moją pracą. |
| 3.6 | Ostatnio zwykle mniej myślę w pracy, a moją pracę wykonuję prawie mechanicznie. |
| 3.7 | Moja praca stwarza wiele pozytywnych wyzwań. |
| 3.8 | W czasie pracy często czuję się emocjonalnie wyczerpany/a. |
| 3.9 | Z czasem można stracić zaangażowanie w wykonywanie tego typu pracy. |
| 3.10 | Po pracy z reguły mam dość siły, aby zająć się czymś dla własnej przyjemności. |
| 3.11 | Czasami to, co robię w pracy, napawa mnie obrzydzeniem. |
| 3.12 | Po pracy zazwyczaj czuję się wykończony/a i znużony/a. |
| 3.13 | To jedyny typ pracy, jaki wyobrażam sobie, że mogę wykonywać. |
| 3.14 | Zazwyczaj dobrze daję sobie radę z ilością pracy, jaką mam wykonać. |
| 3.15 | Z czasem coraz bardziej angażuję się w swoją pracę. |
| 3.16 | Kiedy pracuję, zazwyczaj czuję się pełny/a energii. |

**Jak bardzo zaniepokojony jesteś? (HADS)**

| 4.1 | Czuję się napięty/a lub poddenerwowany/a. |
| --- | --- |
| 4.2 | Wciąż cieszą mnie rzeczy, które zwykle sprawiały mi radość. |
| 4.3 | Odczuwam przerażające uczucie, jakby miało się zdarzyć coś okropnego. |
| 4.4 | Potrafię się śmiać i dostrzegać zabawną stronę zdarzeń. |
| 4.5 | Nachodzą mnie smutne myśli. |
| 4.6 | Czuję się wesoły/a i pogodny/a. |
| 4.7 | Mogę siedzieć spokojnie i czuć się zrelaksowany/a. |
| 4.8 | Czuję się jakbym był/a w "psychicznym dołku" |
| 4.9 | Mam zatrważające uczucie, jakby mi się coś trzęsło w środku. |
| 4.10 | Przestałem/am interesować się swoim wyglądem zewnętrznym. |
| 4.11 | Nie mogę spokojnie usiedzieć na miejscu. |
| 4.12 | Oczekuję z radością na różne sprawy. |
| 4.13 | Miewam nagłe uczucie panicznego lęku. |
| 4.14 | Mogą cieszyć się dobrą książką, programem w radio lub telewizji. |

**Dodatkowe komentarze (Any other comments)**

| 5.1 | Czy jest coś, co chciał(a)byś dodać lub skomentować, szczególnie w związku ze zmianą rodzaju wykonywanej pracy, bezpieczeństwem w pracy lub dob |
| --- | --- |

**APPENDIX C. SAQ SCORING**

| **Scale** | **2020 Questions** |
| --- | --- |
| Teamwork climate | 1,2*,3,4,5,6 |
| Safety Climate | 7,8,9,10,11*,12,13 |
| Job satisfaction | 15,16,17,18,19 |
| Stress recognition | 20,21,22,23 |
| Perceptions of management | 24,25,26,27,28 |
| Working conditions | 29,30,31,32 |
| Overall | All subscale questions, 14, 33, 34, 35, 36* |

*negatively scored, §excluded for the purposes of matching
